# Polymorphisms in melatonin synthesis pathways: possible influences on depression

**DOI:** 10.1186/1740-3391-9-8

**Published:** 2011-08-09

**Authors:** Daniel F Kripke, Caroline M Nievergelt, Greg J Tranah, Sarah S Murray, Michael J McCarthy, Katharine M Rex, Neeta Parimi, John R Kelsoe

**Affiliations:** 1 Department of Psychiatry, University of California, San Diego, 9500 Gilman Drive, La Jolla, California 92093, USA; 2 Viterbi Family Sleep Center, Scripps Clinic, 10666 North Torrey Pines Road, La Jolla, California 92037, USA; 3 Research Institute, California Pacific Medical Center, 185 Berry Street, Suite 5700, San Francisco, California 94107-1728, USA; 4 Scripps Genomic Medicine, Scripps Health, 3344 North Torrey Pines Court, Suite 300, La Jolla, California 92037, USA; 5 Department of Psychiatry, San Diego VA Healthcare System, 3350 La Jolla Village Drive, San Diego, California 92093

**Keywords:** ASMT, N-acetylserotonin, AANAT, melatonin, serotonin, depression, bipolar disorder, lithium

## Abstract

**Background:**

It has been reported that rs4446909, a single nucleotide polymorphism (SNP) in the promoter of acetylserotonin methyltransferase (*ASMT*), influences the expression of the ASMT enzyme. The common G allele is associated with lower ASMT activity, and therefore, diminishes conversion of N-acetylserotonin to melatonin. The G allele was associated with recurrent depressive disorder in a Polish group. ASMT might also affect bipolar relapse, given evidence that N-acetylserotonin might stimulate TRKB receptors, and TRKB may influence mood relapse in bipolar disorder. Additionally, arylalkylamine N-acetyltransferase (*AANAT*) polymorphisms have been reported associated with depression, perhaps through their influence upon N-acetylserotonin or melatonin synthesis.

**Results:**

To replicate and further explore these ideas, rs4446909 was genotyped in four research groups, as part of a panel of 610 SNPs surveyed by an Illumina Golden Gate assay. In 768 cases with delayed sleep phase disorder or matched controls, rs4446909 was indeed associated with the depressive symptoms on a self-report scale (P = 0.01, R^2 ^= 0.007). However, there was no significant association of rs4446909 with self-reported depression in a sleep clinic patient group or with two groups of elderly men and women from multicenter studies, nor was the response to lithium treatment associated with rs4446909 in bipolar patients. No associations of two *AANAT *SNPs with depression were found.

**Conclusions:**

The evidence did not support a strong influence of rs4446909 upon mood, but the partial replication may be consistent with a modest effect. It is possible that larger or younger subject groups with improved phenotype ascertainment might demonstrate more persuasive replication.

## Background

The psychiatric literature contains a number of inconsistent studies of melatonin in affective disorders, reporting that there is a "low melatonin syndrome" or high morning melatonin associated with depression, or that the timing of melatonin secretion may be either advanced or delayed [1-7]. The circadian phase of melatonin secretion has been hypothesized to be a causal element in affective disorders. New evidence suggests that late melatonin elevations may suppress pars tuberalis TEF, a photoperiodic switch which might control human depression [8]. Such a mechanism would be consistent with apparent comorbidity of delayed sleep phase disorder and depression [9]. The problems contributing to inconsistent theories about melatonin's role in depression include the difficulties of obtaining complete overnight melatonin secretion profiles from onset to offset, especially in very disturbed patients, assay difficulties, differences between the home and hospital environments, effects of medications, and the heterogeneity of patient samples.

Since affective disorders appear strongly related to genetic susceptibilities [10], there is hope that modern genetics may clarify the pathological mechanisms in production of melatonin associated with affective disorders. However, the notorious risks of false-positive and non-generalizable reports of genetic association make critical replication essential [11].

**Figure 1:**
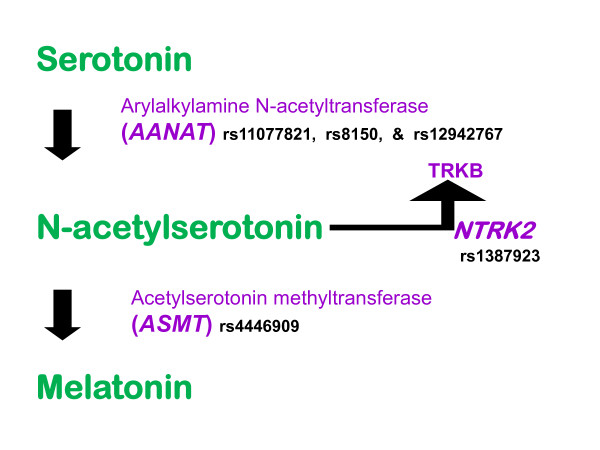
**The metabolic pathway converting serotonin to melatonin involves the enzymes AANAT and ASMT (previously known as HIOMT)**. Although AANAT has been believed to control the rate-limiting step, recent studies [13] imply an effect of ASMT upon the metabolic conversion of serotonin to melatonin. The activity of both AANAT and ASMT might influence the concentration of N-acetylserotonin, which influences expression of TRKB (a BDNF receptor).

Galecki et al. reported that a single nucleotide polymorphism (SNP) in acetylserotonin methyltransferase (*ASMT*) was associated with depression in a Polish group of 181 patients with recurrent depressive disorders contrasted to 149 controls [12]. Being a homozygote for the less common A allele of the rs4446909 SNP was protectively associated with a much lower risk of recurrent depression (odds ratio = 0.1; 95% CI = 0.03-0.58; P = 0.007), whereas the common G allele was associated with more depression. One or two G alleles were observed in 99% of cases and 92% of controls. An additional nearby *ASMT *polymorphism, rs5989681, came close to significant association with recurrent depressive disorders. Furthermore, *ASMT *RNA extracted from whole blood was lower among depressed patients than among controls, and was lower among those with the GG genotype for rs4446909. Since ASMT converts N-acetylserotonin to melatonin (Figure 1), the Polish data were interpreted as consistent with a hypothesized low melatonin syndrome in depression [1].

Previously, Melke and colleagues had reported that the G alleles in these same polymorphisms, rs4446909 and rs5989681, reduced *ASMT *transcription and resulted in low melatonin among autistic children [13]. The G alleles also lowered ASMT activity in unaffected individuals [13]. These polymorphisms evidently alter transcription factor binding sites in the *ASMT *promoter [14]. A microduplication in *ASMT *may also be common in autism [15] though this is unclear [16].

More recently, Soria et al. reported that 3 SNPs in *AANAT*, rs3760138, rs4238989, and rs8150 were modestly associated with depression in a Spanish sample, and the global significance of contrasting haplotypes of these SNPs was P = 6.9 × 10**^-34 ^**[17]. They could not report replication of the associations of these three SNPs in other studies, but a fourth SNP, rs11077820, was significantly associated with depression both in their study and in one subsample of an independent study [17]. The effects of these SNPs on conversion of serotonin to N-acetylserotonin have not been investigated.

When ASMT activity is reduced and less melatonin is produced, N-acetylserotonin might accumulate to higher concentrations (Figure 1), which might explain the observation of increased serotonin (the precursor of N-acetylserotonin) among subjects with reduced ASMT activity [13]. However, in animal models, N-acetylserotonin has an antidepressant-like action [18]. Alterations in the activity of AANAT might also modulate N-acetylserotonin, though regulation of AANAT activity may be largely post-transcriptional [19]. These ideas seem relevant to a recent report that N-acetylserotonin activates neurotrophic tyrosine kinase receptor, type 2 (TRKB), a brain-derived neurotrophic factor (BDNF) receptor transcribed from the *NTRK2 *gene. BDNF binding to TRKB may modulate neuronal survival, neurogenesis, and synaptic plasticity [18,20-22]. Thus, a polymorphism reducing *ASMT *RNA expression and protein activity, by increasing accumulation of N-acetylserotonin, might potentiate actions of TRKB and BDNF. This may be relevant to clinical interests, since colleagues from Dr. Kelsoe's group have shown that the rs1387923 polymorphism near *NTRK2 *is associated with the effectiveness of lithium in preventing relapses of bipolar disorder (many relapses are depressive episodes,) and *NTRK2 *alleles may be associated with suicide [19,23,24]. Theoretically, both *ASMT *and *AANAT *polymorphisms might modulate responsiveness to lithium through TRKB.

With an interest in extending and replicating the recent associations of *AANAT *and *ASMT *polymorphisms with depression, we examined the influence of SNPs in these genes on depression, the circadian trait of morningness-eveningness, and lithium-treatment response in groups of research participants in which our teams had already assayed certain *ASMT *and *AANAT *SNPs.

## Methods

### Participants

This study utilized previous assays of DNA from five research studies (Table 1). All data were collected with written informed consent, approved by the review boards of the participating institutions.

**Table 1:** Studies Included

Study	Participants	N	% Male	Median age (range)	Diagnoses
**Delayed sleep phase disorder**	Cases and Controls	768	33%	35 (22-82)	DSPD or no DSPD

**Sleep Clinic**	Scripps sleep disorders patients	853	65%	57 (21-96)	Sleep apnea, DSPD, insomnia, etc.

**MrOS**	Volunteers, multi-center	2540	100%	76 (67-96)	Mixed diagnoses or none

**SOF**	Volunteers, multi-center	1731	0%	83 (79-99)	Mixed diagnoses or none

**Bipolar disorder**	VA clinic patients	80	63%	46 (21-67)	All had bipolar disorder

First, 768 participants in a study of the genetics of delayed sleep phase disorder (DSPD) were about half cases and about half ancestry-matched controls, with the majority of European ancestry [9]. Their ages ranged from 22 to 82 with a median of 35 years, and 67% were female.

Second, Scripps Clinic Viterbi Family Sleep Center patient volunteers included 853 who underwent diagnostic polysomnography for sleep complaints. Their ages ranges from 21 to 96 years (median 57), 65% were male, and 88% were of European ancestry. At least half of these patients had more than average sleep respiratory disturbance; insomnia, restless legs syndrome, and delayed sleep phase disorders were also common. The UCSD DSPD and Scripps participants each completed the Quick Inventory of Depressive Symptomatology Self-Report questionnaire (QIDS-SR) for evaluation of current-state depression, a scale highly correlated with the gold-standard Hamilton Depression Rating Scale [25]. Although the QIDS-SR score is well correlated with research diagnoses of current major depressive episodes [26] and therefore, is associated with a lifetime diagnosis of recurrent major depressive disorder, it does not always provide an accurate reflection of the lifetime history of depression, since patients may have been more depressed at prior times in their lives. Participants also completed the BALM morningness-eveningness scale, a simplified scale well-correlated with the Horne-Östberg morningness-eveningness scale for measuring circadian traits [9,27].

Third, in the Osteoporotic Fractures in Men (MrOS) Study [28,29], 5,994 ambulatory community-living males at least age 65 years at study entry were recruited at 6 clinical centers in the U.S. (Birmingham, AL, Minneapolis, MN, Palo Alto, CA, Pittsburgh, PA, Portland OR, and San Diego, CA.) This analysis included 2540 participants self-reporting white race who had given DNA samples, and who 3.5 years after entry at ages ranging from 67-96 years (median 76) participated in a MrOS Sleep ancillary study (Outcomes of Sleep Disorders in Older Men), when they completed the Geriatric Depression Scale (GDS) [30]. Like the QIDS-SR, the GDS is no exact measure of lifetime depression susceptibility, but log_10_[1+GDS] was used as an indicator of current depression state in the geriatric age group.

Fourth, the Study of Osteoporotic Fractures (SOF) is a study focusing on community-dwelling Caucasian women over age 65 years recruited at 4 centers: The University of Maryland (Baltimore), the University of Minnesota, the University of Pittsburgh, and The Kaiser Permanente Center for Health Research (Portland) [31]. From an original cohort of 9,704 elderly women, 2431 with available DNA were genotyped as part of a study of circadian genes and sleep, of which 1,786 attended visit 8 where the GDS was ascertained. At this point, they were from 79-99 years of age (median 83 years.) Included in the study were 1,731 women with genotype data, after exclusion of those with missing values for GDS.

Fifth, VA San Diego Health Care System participants were patients with bipolar affective disorder receiving lithium to prevent relapse. Patients with bipolar disorder were interviewed using the Diagnostic Interview for Genetic Studies (DIGS). As part of this interview, they were queried regarding past medication trials using a life chart method. Information from the patient, medical records and family informants were considered by a panel of blind clinical raters. All trials of lithium were considered, and episode frequency on and off lithium were compared. Greater weighting was allowed for monotherapy trials. Response was scored as *good *for 50% reduction in episodes and *not good *for those with less or no response. These data were also used to rate lithium response using the Alda scale, which scores response on a 1-10 scale and corrects for poor information or compliance [32]. Analyses were restricted to 80 bipolar patients of European ancestry to avoid stratification artifacts. Their ages ranged from 21 to 67 years (median 46), and 63% were male.

### Assay Methods

The CPMC investigators, the UCSD group, and the Scripps Clinic researchers had collaborated to develop an assay for polymorphisms in circadian and sleep-related genes. A custom Illumina Golden Gate assay was developed to genotype 768 SNPs, of which 610 were found to yield heterozygous alleles in Hardy-Weinberg equilibrium and adequate assay quality, e.g., as judged by 99.4% genotype calling. This assay included 12 SNPs in *ASMT *and its promoter regions, including rs4446909, SNPs that were selected for a combination of possible functional roles and tagging considerations. An additional 2 *ASMT *region SNPs were imputed using the program MACH v. 1.0.16 [33]. Phased haplotypes of 60 unrelated HapMap II CEU founders were used as the reference data (CEU_r21_nr_fwd_phased). Unfortunately, rs5989681 (another *ASMT *promoter SNP implicated in depression) was not assayed directly nor imputed successfully. An additional 29-41 continental-ancestry-informative marker SNPs (AIMs) were assayed in the UCSD DSPD, Scripps and bipolar disorder DNA, to exclude participants of non-European ancestry, and the MrOS and SOF participants were all known to be of European ancestry. Three *AANAT *SNPs, rs11077821, rs8150, and rs12942767 were also assayed in the five groups. Each bipolar patient had been genotyped for *NTRK2 *rs1387923 using a Taqman assay.

### Statistical Analysis

The associations of *ASMT *SNPs with either the QIDS-SR or GDS depression scales were computed with PLINK [34], using linear regression in an additive model, controlling for age, gender (when applicable), and clinic site (when applicable). To control for ancestry background, we performed a multidimensional scaling analysis (MDS) with the AIMs and used two ancestry-informative marker dimensions as covariates in the association analyses. Because we were seeking to replicate the previous reports of Galecki et al. [12] and Soria et al. [17] of SNPs associated with depression, a significance criterion of P = 0.05 was chosen for *ASMT *rs4446909 and *AANAT *rs8150. R^2 ^represented the percentage of depression variance predicted by the SNP, excluding covariates from the model. For rs4446909, a recessive model was also considered, but it did not improve the evidence for association of the SNP with depression. Association of *ASMT *and *AANAT *SNPs with BALM was examined in the UCSD and Sleep Center participants with a dominant model, using similar linear regressions controlled for age, gender, and MDS factors, and correcting for multiple testing of 610 SNPs. The dominant model was selected because of evidence that morningness-eveningness is inherited as a dominant trait [35]. Among the bipolar patients, the associations of *ASMT *rs4446909 and *NTRK2 *rs1387923 with lithium response and Alda scale response were assessed with SPSS, using the general linear model and backwards stepwise logistic regression, controlling for age and gender.

## Results

Results of the regression analyses are summarized in Table 2. As predicted, rs4446909 was significantly associated with depressive symptoms (QIDS-SR) in the UCSD DSPD cases and controls (P < 0.01, R^2 ^= 0.007). The association of rs4446909 with depressive symptoms in the Sleep Center patients was not significant (P = 0.10, R^2 ^= 0.004). Likewise, in the MrOS participants, the association of rs4446909 with the GDS was not significant (P = 0.06, R^2 ^= 0.0003), nor was it significant among SOF participants (P = 0.26, R^2 ^= 0.0006). Exploration of *ASMT *haplotypes using sliding windows of 2, 3, or 4 SNPs [34] did not reveal additional significant associations.

**Table 2:** Association of *ASMT *rs4446909 with depression scales.

Study	rs4446909 P	rs4446909 R^2^	rs4446909 Beta
**DSPD**	0.01	0.007	-0.74

**Sleep Clinic**	0.10	0.004	0.40

**MrOS**	0.06	0.0003	0.02*

**SOF**	0.26	.0006	0.01*

P: nominal values, corrected for genomic inflation, are for additive association of the minor allele of rs4446909 with the QIDS or GDS depression self-rating. Recessive models were not more significant.

R^2^: squared correlation representing percent variance explained by the association of the depression score and rs4446909, uncorrected for covariates.

Beta: for DSPD, a negative beta indicated that as the A allele decreases in amount (frequency), and G alleles increase, QIDS depression scores increase. The not-significant Beta's for the Sleep Clinic, MrOS, and SOF participants were in the opposite direction from the DSPD sample.

* Beta for the log transform of GDS+1 suggested a 2% increase in GDS score over the range of 1 to 16 per A allele.

Only 3 SNPs in *AANAT *were assayed, rs8150, rs11077821, and rs12942767. None of these were significantly associated with the QIDS-SR or log_10_[GDS] in any of the four research groups rated for depression.

The same analyses could not be done for the bipolar disorder patients, for whom depression scales were not collected. However, it was of interest that the rs4446909 minor allele frequency of 0.24 - 0.27 was similar in the UCSD DSPD, Sleep Center, MrOS, SOF, and bipolar DNAs, suggesting no association of the rs4446909 allele with bipolar disorder. In the analysis of bipolar patients, neither the categorical lithium response nor the Alda score were significantly associated with either rs4446909 or rs1387923, controlling for age and gender, nor were any significant interactions observed.

In regard to the BALM morningness-eveningness scale, no *ASMT *SNP was significantly associated in the UCSD DSPD or Sleep Center participants, controlling for age and gender. BALM scores were not available for the MrOS and SOF participants. Among the bipolar patients, the BALM was nominally significantly associated with rs4446909, controlling for rs1387923, age and gender in ANOVA models (P = 0.009), but there were no significant interactions. The minor A allele was associated with a higher BALM score, indicating morning preference. However, this association was not significant when correcting for the multiple testing of 610 SNPs, which had been done for testing association with the BALM. Combining the DSPS and Sleep Clinic samples, which was justified by the distribution of the BALM in the samples, *AANAT *rs11077821 was nominally associated with the BALM (A allele associated with greater BALM, i.e., greater morningness) with P = 0.019, a P value which did not survive Bonferroni correction for multiple testing. No significant interaction of *AANAT *rs11077821 with *ASMT *rs4446909 was observed affecting the BALM or QIDS-SR in the combined DSPS and Sleep Clinic samples, nor were any *ASMT *or *AANAT *SNPs associated with lithium response in the bipolar sample, nor were interaction effects between such SNPs observed.

## Discussion

These studies yielded both significant and non-significant results, some of which suggest inability to replicate previously-reported findings. To summarize, we were able to replicate an association of the *ASMT *rs4446909 G allele with depressive symptoms in one of the four groups examined (P < 0.01, a P value which survives Bonferroni correction for four groups tested). In the DSPD study participants, a negative regression indicated that as the rare A allele decreased in frequency and the common G allele increased, depressive symptoms measured by the QIDS-SR score tended to increase, consistent with the association previously reported by Galecki et al. [12]. In the Sleep Center, MrOS, and SOF participants, rs4446909 was not associated with depression symptoms at the P < 0.05 level. Neither was rs4446909 associated with lithium response among bipolar participants. The R^2 ^values were not large in any of the groups, suggesting that *ASMT *would not appear to be an important factor in the expression of depressive symptoms or the stability of lithium-treated bipolar disorder. Possibly a stronger influence of *ASMT *polymorphisms might be demonstrated in younger populations, using different phenotyping methods. We could not replicate an association of *AANAT *rs8150 with depression in any of the 4 groups, though we have not assayed the other two SNPs necessary to examine the strongly-associated haplotype reported by Soria et al. [17]. Also, we have not assayed rs11077820, the one imputed SNP in the Spanish subjects which was replicated in another study, though we had assayed rs11077821, a nearby SNP only 911 nucleotides 3' which was significantly associated with depression in the WTCCC study [17] but not in our studies. We did observe nominally significant associations of *ASMT *rs4446909 and *AANAT *rs11077821 with the BALM morningness-eveningness scale, implying an influence of these SNPs upon circadian phase, but since we tested a large number of SNPs for these associations which were not predicted prospectively, they did not meet Bonferroni significance criteria correcting for multiple testing.

A limitation of our methods was the use of self-report depression scales which reflected the recent mood state of the subject, rather than any specific lifetime diagnosis, whereas the Polish and Spanish depression cases were all diagnosed with major depressive disorders by accepted criteria. The methods of Galecki et al. [12] and Soria et al. [17] may have better distinguished lifetime genetic susceptibility to depressive disorders from evidence of no susceptibility, since some of our research participants with high depressive symptom scores may not have suffered recurrent depression, whereas some patients with low scores at the time of testing may have suffered episodes of depression in the past. Also, many of the Sleep Center patients and all of the MrOS and SOF participants were quite elderly, and it is known that genetic susceptibility to depression may be less influential when cerebrovascular disease and various forms of dementia begin to alter the depressive phenotypes [36,37].

Direct clinical evidence for low melatonin as a cause of depression has been quite inconsistent. Our group has not found evidence associating low melatonin with major depression [4], but rather our evidence favored a delayed offset of the nocturnal melatonin peak, extending after awakening [3], which might be consistent with the recent report indicating that delayed melatonin offset inhibits photoperiodic responses [8]. Indeed, in our previous data, there was some trend associating high melatonin release with depression [4]. Similar findings of delayed melatonin offset in depression have been reported by others [6,38]. If the G allele of *ASMT *rs4446909 tends to reduce ASMT expression and activity, that might increase the half-life of N-acetylserotonin and thereby delay the synthesis and subsequent metabolism of melatonin, thus delaying both the onset and offset of the nocturnal melatonin peak and delaying the melatonin phase. The G allele was likewise associated with delayed circadian phase as measured by the BALM (not significant after correction for multiple testing). A reduction of ASMT activity might have an impact on the timing of melatonin synthesis independent of its overall 24-hour production. Factors influencing the sequestration of AANAT might likewise influence melatonin timing.

Another possibility, perhaps implied by increased serotonin associated with autism spectrum disorder and presumably with the rs4446909 G allele [13], is that a crucial effect of reduced ASMT expression is accumulation of N-acetylserotonin, which we now appreciate may influence the BDNF-TRKB interaction [18]. It is possible that certain alleles of *AANAT *SNPs would also increase concentrations of N-acetylserotonin. We could not demonstrate a clinical effect in the bipolar patients utilizing only 80 patients, but larger groups were needed for the previous studies to demonstrate an effect of the rs1387923 SNP in *NTRK2*. Further exploration of *ASMT *and *AANAT *SNPs in larger pharmacogenetic studies of *NTRK2 *and lithium should be encouraged. Although psychiatry has focused primarily on serotonin and melatonin, it has been known for decades that N-acetylserotonin might play an independent role [39].

We have focused on the last two enzymes in melatonin synthesis, but numerous other genes influence the transcription and sequestration of these enzymes, as well as melatonin metabolism [19,40]. Among these, *MAOA*, *COMT, TPH1*, and *TPH2 *have been reported to be associated with depression, but a recent genome-wide association study found no significant genome-wide association, and the best candidates did not involve these pathways [41]. MAOA is of particular interest, both because there are several positive reports of association, and because MAOA may participate in regulating both in synthesis and degradation of melatonin [42,43].

## Conclusions

To summarize, our data from five research samples do not demonstrate any strong association of *AANAT *and *ASMT *SNPs with depression, though a weak association with rs4446909 was replicated. We regard the overall evidence of our group and others as sufficiently promising to invite additional more detailed studies, especially when full sequencing of these genes in clinical samples becomes practical. Additional understanding of melatonin synthesis pathways may demonstrate a more important contribution to depression.

## Competing interests

The authors declare that they have no competing interests.

## Authors' contributions

DFK conceived the manuscript, wrote much of it, and initiated data collection for the DSPD and Scripps studies. CMN developed the circadian and AIMs SNP panels and consulted on all statistical analyses. GJT arranged for the genotyping of the MrOS and SOF samples and supervises analyses of these genotypes. SSM called the genotypes for all assays. MJM helped recruit and analyze the bipolar patients. KMR supervised recruitment of the DSPD participants and scored phenotypes. NP did statistical analyses of MrOS data and SOF. JRK initiated investigation of the bipolar patients and supervised some of the laboratory assays. All authors contributed to the manuscript.
